# Anterior Nutcracker Syndrome: First Surgically Managed Case Reported in Benin

**DOI:** 10.1055/a-2699-8210

**Published:** 2025-09-24

**Authors:** Abdel Kémal Bori Bata, Joseph Akodjenou, Ahmad Ibrahim, Boris Gogan, Désiré Nékoua

**Affiliations:** 1 University Visceral Surgery Clinic (CNHU-HKM), Faculty of Health Sciences, University of Abomey-Calavi, Cotonou, Benin; 2 University Cardiology Clinic (CNHU-HKM), Faculty of Health Sciences, University of Abomey-Calavi, Cotonou, Benin; 3 Anesthesia and Intensive Care Unit, CHU-MEL, Faculty of Health Sciences, University of Abomey-Calavi, Cotonou, Benin; 4 University Cardiology Clinic (CNHU-HKM), Faculty of Health Sciences, University of Abomey-Calavi, Cotonou, Benin; 5 University Pediatric Surgery Clinic, CNHU-HKM, Faculty of Health Sciences, University of Abomey-Calavi, Cotonou, Benin; 6 Multipurpose University Clinic of Anesthesia and Resuscitation (CNHU-HKM), Faculty of Health Sciences, University of Abomey-Calavi, Cotonou, Benin

**Keywords:** nutcracker syndrome, varicocele, left renal vein transposition, sub-Saharan Africa

## Abstract

**Background:**

Nutcracker syndrome is a rare condition caused by compression of the left renal vein, often underdiagnosed in adolescents.

**Case Description:**

We report a 16-year-old male with painful left testicular swelling and grade 3 varicocele. Imaging confirmed anterior nutcracker syndrome. He underwent successful left renal vein transposition with complete symptom resolution and normal imaging at 24 months.

**Conclusion:**

Nutcracker syndrome should be considered in young patients with varicocele and testicular pain. Renal vein transposition is a safe and effective treatment when conservative approaches fail, with long-term clinical and radiological benefits.

## Introduction

Nutcracker syndrome (NCS) refers to clinical symptoms caused by extrinsic compression of the left renal vein (LRV). 1 2 It exists in two anatomical variants: anterior nutcracker syndrome (ANCS), where the LRV is compressed between the superior mesenteric artery (SMA) and the aorta, and posterior NCS, where a retroaortic LRV is compressed between the aorta and the vertebral column. 3 The clinical spectrum is highly variable and diagnosis typically requires clinical and radiological evidence of LRV compression. 2 4

Although frequently benign, symptoms can be severely debilitating, necessitating surgical intervention in selected cases. 1 We present the first documented surgical case of ANCS in Benin, managed via LRV transposition with favorable postoperative outcomes.

## Case Presentation

**Fig. 1:**
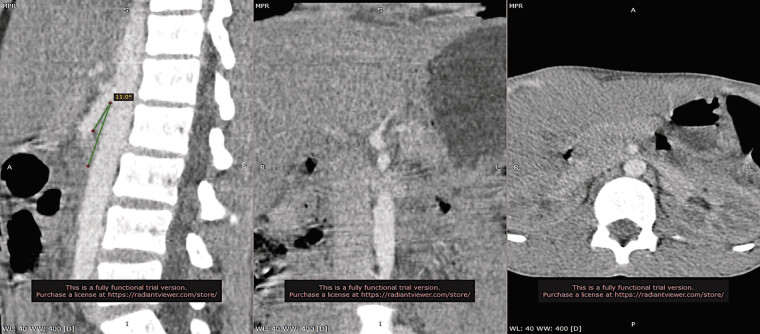
Contrast-enhanced computed tomography (CT) images showing anterior nutcracker syndrome. The sagittal view demonstrates a narrowed aortomesenteric angle, and the axial view reveals the classic “beak sign” due to compression of the left renal vein (LRV).

A 16-year-old previously healthy male with a low body mass index (BMI) of 16.49 kg/m ^2^ presented with a 7-month history of left testicular pain exacerbated by physical exertion. Examination revealed a tender, visible varicosity of the anterior pampiniform plexus and microscopic hematuria without proteinuria. Scrotal Doppler ultrasound demonstrated left testicular hypotrophy and a grade 3 varicocele, with left-sided pampiniform plexus dilation evident in the supine position and accentuated in the upright position. Abdominal Doppler ultrasonography performed in both supine and upright positions showed near-complete narrowing of the LRV at the aortomesenteric clamp, with a characteristic “beak sign.” The hilar-to-aortomesenteric LRV diameter ratio was 8, and the peak velocity ratio between the aortomesenteric and hilar portions was 5. No dilation or abnormal flow was noted in the inferior vena cava (IVC) or iliac veins. Contrast-enhanced abdominal computed tomography (CT) angiography ( Fig. 1 ) confirmed the diagnosis of ANCS, revealing an SMA aortic angle of 11 degrees, the “beak sign,” and a hilar-to-aortomesenteric LRV diameter ratio of 6.5. The left gonadal vein was dilated. Renal function tests were within normal limits. Initial conservative management consisted of analgesics and nutritional support aimed at weight gain, administered over 4 months. Symptoms worsened with pelvic pain and persistent microscopic hematuria.

**Fig. 2:**
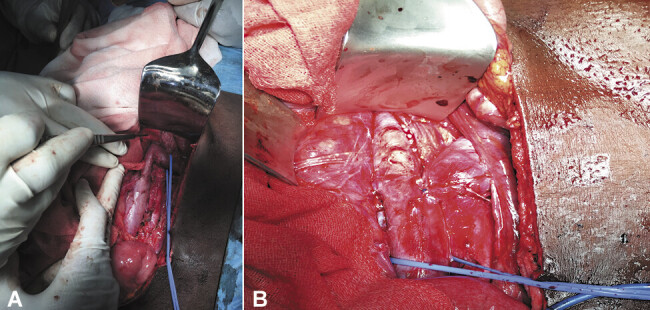
Intraoperative images of left renal vein (LRV) transposition onto the inferior vena cava (IVC). ( **A** ) Initial anatomical insertion site of the LRV before transposition. ( **B** ) Reimplanted LRV anastomosed to the IVC, 3-cm inferior to its original insertion site.

The patient underwent surgery via a midline supra- and infraumbilical laparotomy under general anesthesia. Following systemic heparinization, lateral clamping of the IVC was performed, and the LRV was transected and reimplanted 3-cm caudal to its original insertion ( Fig. 2 ). Postoperative anticoagulation with enoxaparin was administered for 72 hours. The postoperative course was uneventful. Immediate Doppler ultrasound confirmed satisfactory LRV patency without thrombosis, distortion, or stenosis. The patient was discharged on postoperative day 8.

At the 24-month follow-up, the patient reported no recurrent pain or testicular swelling. Computed tomography angiography (CTA) showed no residual compression of the LRV by the SMA, nor any evidence of intrinsic stenosis.

## Discussion

Initially described anatomically by Grant in 1937 and later identified in humans by El Sader and Mina in 1950, the term “nutcracker syndrome” was coined by de Schepper in 1972. Its true prevalence remains unknown due to frequent asymptomatic presentation and diagnostic ambiguity. It typically affects young to middle-aged adults, with a slight female predominance (53.6%). 1 4 Several studies have reported an association between BMI and NCS. 1 Our patient, with a BMI of 16.49 kg/m ^2^ , supports this correlation.

NCS is often a diagnosis of exclusion. 2 Common presenting symptoms include microscopic hematuria (69.5%), flank or abdominal pain (48.4%), pelvic pain (23.1%), and varicocele (15.8%) 2 —all of which were present in our patient.

ANCS diagnosis relies on multiple imaging modalities, including Doppler ultrasonography, CTA, magnetic resonance angiography (MRA), intravascular ultrasound, and venography with pressure gradient measurement. 1 2 4 Doppler ultrasonography is the first-line imaging modality for diagnosing nutcracker syndrome (NCS), due to its accessibility, safety, and real-time hemodynamic assessment. The key parameter is the peak systolic velocity ratio between the aortomesenteric and hilar segments of the LRV. Diagnostic thresholds are ≥5.0 in adults (80% sensitivity, 94% specificity) and ≥4.7 in children (100% sensitivity, 90% specificity). Accurate measurement requires optimizing the insonation angle (ideally 30–60 degrees) and adjusting sample volume to fully include the SMA segment. Color Doppler enhances diagnostic confidence by visualizing aliasing high-velocity jet flows. A Doppler-derived pressure gradient > 3 mm Hg is considered pathognomonic. 5 CTA and MRA are valuable for visualizing LRV compression, gonadal vein dilation, and pelvic congestion. A hilar-to-aortomesenteric LRV diameter ratio >4.9, “beak sign,” and an SMA–aortic angle <35 degrees are highly suggestive. 2 In our case, combined Doppler ultrasound and CTA enabled accurate diagnosis, in line with existing literature. 4 Furthermore, CTA was selected over MRA despite its ionizing nature, due to the unavailability of magnetic resonance imaging in our setting.

Management of ANCS is debated due to the absence of consensus guidelines. Therapeutic decisions depend on symptom severity. Conservative management is favored in patients with mild symptoms (e.g., microscopic hematuria, orthostatic proteinuria), particularly those under 18, as growth may alleviate LRV compression through increased retroperitoneal fat and connective tissue. 1 2 This approach includes diet (notably weight gain), analgesia, and clinical monitoring. Its aim is to relieve clinical symptoms and prevent potentially severe complications such as chronic anemia or thrombosis of the LRV. Wang et al. reported favorable outcomes with conservative therapy in two-thirds of patients. 6 Similarly, Sarikaya et al., in a prospective study involving 16 patients with moderate symptoms followed for a median duration of 27.3 months, observed complete symptom resolution in 28.5% of cases and improvement in 31.4%, despite the absence of significant changes in imaging parameters. 7 Surgical intervention is reserved for refractory or severe cases and can be performed via open or minimally invasive approaches. In our patient, surgery was warranted due to persistent disabling pain after 4 months of conservative therapy and the infertility risk linked to varicocele. This risk is supported by studies showing impaired spermatogenesis in NCS-related varicocele and significant fertility improvement after microsurgical spermatic–inferior epigastric vein anastomosis, with natural conception achieved in 80% of cases. 8

Among available surgical options, open approaches—particularly LRV transposition—are still regarded as the reference standard for the definitive management of ANCS. Other procedures such as nephropexy, renal autotransplantation, or gonadocaval bypass have also been described, but with more limited data. LRV transposition has shown durable results, with a low incidence of major perioperative complications and a favorable safety profile. In the Mayo Clinic series, there were no reported cases of renal failure or mortality, and early symptom recurrence was uncommon (8.3%), mainly due to LRV restenosis or occlusion, which were effectively managed by secondary interventions. Over a mean follow-up of 36.8 months, the reintervention rate reached 22.2%, while patency rates at 24 months remained high (74% primary, 97% primary-assisted, 100% secondary). 2 3

Endovascular techniques, such as LRV stenting and gonadal vein embolization, are less invasive and preserve renal perfusion with faster recovery. However, their long-term safety—especially in younger patients—remains uncertain. Complications like migration (up to 6.6%), fracture, or occlusion may require surgical retrieval. Despite over 90% technical success and symptom relief, these risks, along with the lack of dedicated venous stents and limited follow-up, restrict their use in pediatric populations. 2 4

Laparoscopic extravascular stenting is a promising minimally invasive option, offering direct operative control without LRV clamping. Small series report favorable hemodynamics and symptom relief, although evidence remains limited and rare complications, such as stent migration, have been noted. 2

In our patient, the absence of symptoms 2 years after surgery, along with preserved renal function and no radiological evidence of residual compression, aligns with the existing literature and further supports the feasibility and efficacy of LRV transposition in resource-limited setting.

## Conclusion

Nutcracker syndrome is a rare and often underdiagnosed vascular compression disorder with variable clinical presentations. While multiple therapeutic options exist, open surgical transposition of the LRV remains the gold standard in selected cases. This case highlights the efficacy and safety of open LRV transposition in sub-Saharan African settings and adds to the global experience of nutcracker syndrome management.

## References

[1] Ananthan K Onida S Davies A H Nutcracker syndrome: an update on current diagnostic criteria and management guidelines Eur J Vasc Endovasc Surg 2017 53 06 886 894 28356209 10.1016/j.ejvs.2017.02.015

[2] Velasquez C A Saeyeldin A Zafar M A Brownstein A J Erben Y A systematic review on management of nutcracker syndrome J Vasc Surg Venous Lymphat Disord 2018 6 02 271 278 29292117 10.1016/j.jvsv.2017.11.005

[3] Ali-El-Dein B Osman Y Shehab El-Din A B El-Diasty T Mansour O Ghoneim M A Anterior and posterior nutcracker syndrome: a report on 11 cases Transplant Proc 2003 35 02 851 853 12644163 10.1016/s0041-1345(02)04026-5

[4] Nastasi D R Fraser A R Williams A B Bhamidi V A systematic review on nutcracker syndrome and proposed diagnostic algorithm J Vasc Surg Venous Lymphat Disord 2022 10 06 1410 1416 36007798 10.1016/j.jvsv.2022.08.003

[5] Kim S H Doppler US and CT diagnosis of nutcracker syndrome Korean J Radiol 2019 20 12 1627 1637 31854150 10.3348/kjr.2019.0084 PMC6923211

[6] Wang L Yi L Yang L Diagnosis and surgical treatment of nutcracker syndrome: a single-center experience Urology 2009 73 04 871 876 19193424 10.1016/j.urology.2008.11.043

[7] Sarikaya S Altas O Ozgur M M Outcomes of conservative management in patients with nutcracker syndrome Phlebology 2024 39 06 403 413 38452734 10.1177/02683555241238772

[8] Li H Zhang M Jiang Y Zhang Z Na W Microsurgical spermatic-inferior epigastric vein anastomosis for treating nutcracker syndrome-associated varicocele in infertile men: a preliminary experience Urology 2014 83 01 94 99 24207161 10.1016/j.urology.2013.08.050

